# The critical care response to a hospital outbreak of Middle East respiratory syndrome coronavirus (MERS-CoV) infection: an observational study

**DOI:** 10.1186/s13613-016-0203-z

**Published:** 2016-10-24

**Authors:** Hasan M. Al-Dorzi, Abdulaziz S. Aldawood, Raymond Khan, Salim Baharoon, John D. Alchin, Amal A. Matroud, Sameera M. Al Johany, Hanan H. Balkhy, Yaseen M. Arabi

**Affiliations:** 1 ICU2 and TICU, Intensive Care Department, King Abdulaziz Medical City, King Abdullah International Medical Research Center, King Saud bin Abdulaziz University for Health Sciences, Riyadh, Saudi Arabia; 2 Intensive Care Department, King Abdulaziz Medical City, King Abdullah International Medical Research Center, King Saud bin Abdulaziz University for Health Sciences, Riyadh, Saudi Arabia; 3 Neuro-ICU, Intensive Care Department, King Abdulaziz Medical City, King Abdullah International Medical Research Center, King Saud bin Abdulaziz University for Health Sciences, Riyadh, Saudi Arabia; 4 IMCU, Intensive Care Department, King Abdulaziz Medical City, King Abdullah International Medical Research Center, King Saud bin Abdulaziz University for Health Sciences, Riyadh, Saudi Arabia; 5 ICU2, King Abdulaziz Medical City, Riyadh, Saudi Arabia; 6 TICU, King Abdulaziz Medical City, Riyadh, Saudi Arabia; 7 Microbiology Laboratory, King Abdulaziz Medical City, Riyadh, Saudi Arabia; 8 Department of Infection Prevention and Control, King Abdulaziz Medical City, King Saud bin Abdulaziz University for Health Sciences, Riyadh, Saudi Arabia; 9 Intensive Care Department, Respiratory Services, College of Medicine, King Abdulaziz Medical City, King Abdullah International Medical Research Center, King Saud bin Abdulaziz University for Health Sciences, ICU 1425, PO Box 22490, Riyadh, 11426 Saudi Arabia

**Keywords:** Severe acute respiratory infection, Middle East respiratory syndrome, Saudi Arabia, Critical care, Disease outbreak, Disaster planning

## Abstract

**Background:**

Middle East respiratory syndrome coronavirus (MERS-CoV) has caused several hospital outbreaks, including a major outbreak at King Abdulaziz Medical City, a 940-bed tertiary-care hospital in Riyadh, Saudi Arabia (August–September 2015). To learn from our experience, we described the critical care response to the outbreak.

**Methods:**

This observational study was conducted at the Intensive Care Department which covered 5 ICUs with 60 single-bedded rooms. We described qualitatively and, as applicable, quantitatively the response of intensive care services to the outbreak. The clinical course and outcomes of healthcare workers (HCWs) who had MERS were noted.

**Results:**

Sixty-three MERS patients were admitted to 3 MERS-designated ICUs during the outbreak (peak census = 27 patients on August 25, 2015, and the last new case on September 13, 2015). Most patients had multiorgan failure. Eight HCWs had MERS requiring ICU admission (median stay = 28 days): Seven developed acute respiratory distress syndrome, four were treated with prone positioning, four needed continuous renal replacement therapy and one had extracorporeal membrane oxygenation. The hospital mortality of ICU MERS patients was 63.4 % (0 % for the HCWs). In response to the outbreak, the number of negative-pressure rooms was increased from 14 to 38 rooms in 3 MERS-designated ICUs. Patients were managed with a nurse-to-patient ratio of 1:0.8. Infection prevention practices were intensified. As a surrogate, surface disinfectant and hand hygiene gel consumption increased by ~30 % and 17 N95 masks were used per patient/day on average. Family visits were restricted to 2 h/day. Although most ICU staff expressed concerns about acquiring MERS, all reported to work normally. During the outbreak, 27.0 % of nurses and 18.4 % of physicians working in the MERS-designated ICUs reported upper respiratory symptoms, and were tested for MERS-CoV. Only 2/196 (1.0 %) ICU nurses and 1/80 (1.3 %) physician tested positive, had mild disease and recovered fully. The total sick leave duration was 138 days for nurses and 30 days for physicians.

**Conclusions:**

Our hospital outbreak of MERS resulted in 63 patients requiring organ support and prolonged ICU stay with a high mortality rate. The ICU response required careful facility and staff management and proper infection control and prevention practices.

## Background

The Middle East respiratory syndrome (MERS) coronavirus is a recently identified virus that is closely related to the severe acute respiratory syndrome coronavirus (SARS-CoV) [1], causes severe hypoxemic respiratory failure with multiorgan failure and frequently requires admission to the intensive care unit (ICU) [2, 3]. As of September 27, 2016, the World Health Organization (WHO) reported 1806 laboratory-confirmed cases, including 643 related deaths [4]. The majority (~80 %) of MERS cases occurred in Saudi Arabia [4], where several hospital outbreaks happened [5, 6] with 45 % of all cases taking place within healthcare facilities [7]. The outbreak in the Republic of Korea illustrated the global threat of this disease. It started in May 2015 and resulted from a case with travel history to the Arabian Peninsula [4]. Human-to-human transmission occurred to close contacts [family members, other patients and healthcare workers (HCWs)] and led to 186 cases of MERS-CoV infections with 19 % fatality rate. In our hospital, King Abdulaziz Medical City-Riyadh, an outbreak of MERS disease occurred in August to September 2015 [4], led to significant disruption of hospital functions and resulted in 130 MERS cases [8] with 63 patients requiring ICU admission. The outbreak was attributed to crowding, movement of infected but undiagnosed patients and breaches in infection prevention and control practices [4]. Details of the hospital outbreak have been published elsewhere [8].

Most of the medical literature on MERS has focused on describing the characteristics and outcomes of affected patients. Preparedness and the response of the healthcare system at its different levels are crucial to contain this disease and manage its associated outbreaks. Nevertheless, little is published about how the healthcare system responded to the disease and hospital outbreaks. The objective of this study was to describe the response of the ICU to a hospital MERS outbreak, the associated changes in its workflow and the impact on its HCWs.

## Methods

### Setting

This study was an observational study conducted at the Intensive Care Department of King Abdulaziz Medical City, a 940-bed tertiary-care referral hospital in Riyadh, Saudi Arabia, that was accredited by the Joint Commission International. The hospital had an Infection Prevention and Control Department.

The Intensive Care Department covered 5 units: an 8-bed trauma ICU (Unit A), a 21-bed medical-surgical intensive care unit (Unit B), a 9-bed surgical ICU (Unit C), an 8-bed neurologic ICU (Unit D) and a 14-bed stepdown unit (Unit E). The Department also provided coverage to boarding patients in the 15-bed Resuscitation Area in the Emergency Department (ED). The hospital had also an 8-bed Burn ICU. The ICUs were operated as closed units with 24-h, 7-day onsite coverage by board-certified critical care intensivists [9]. Normally, 5 medical teams covered the units during the day with each team consisting of one intensivist consultant, one registrar/fellow and 1–3 residents. The nurse-to-patient ratio in all the ICUs was mostly 1:1. One certified respiratory therapist covered a maximum of six ventilated patients. Additionally, the department had a rapid response team, which covered the hospital wards and was activated according to predefined criteria and is covered by a sixth separate team [10].

The department has had several ongoing quality improvement projects and indicators. Infection prevention and control practices, such as hand hygiene and the ventilator care bundle, were monitored by multidisciplinary ICU teams and the Infection Prevention and Control Department [11]. For intubated patients, ventilator care was provided by specialized respiratory therapists and included using closed endotracheal suctioning systems which were changed every 72 h or as clinically indicated [12], changing the ventilator circuits in between patients or if they became soiled or damaged [12], and using heat and moisture exchangers which were changed every 7 days or when visibly soiled [12]. Due to the high prevalence of multidrug-resistant organisms and prior cases of influenza and MERS infection in the hospital, droplet precautions were applied to all ICU patients since February 2012 [11]. Sporadic cases of MERS cases have been managed in our ICU since February 2013. The characteristics, management and outcomes of the initial MERS cases managed in our unit were described previously [3]. All HCWs were required to undergo fit testing for the N95 respirators (Table 1).

**Table 1:** Selected elements of the Infectious Disease Epidemic Plan of King Abdulaziz Medical City-Riyadh as it relates to the intensive care (initial release in May 2014 and first revision in March 2015)

	Task/description
Plan activation	The plan will be activated by the Chair of the outbreak response committee based on the phase definition *Phase I* 0–5 cases of suspected or confirmed in the hospital *Phase II* 6–30 cases of suspected or confirmed in the hospital *Phase III* **>**30 cases of suspected or confirmed in the hospital
MERS patient placement	*Phase I* Confirmed MERS-CoV cases requiring intubation will be assigned a negative-pressure room and cohorted in one ICU Confirmed cases that have been diagnosed with MERS-CoV in any ICU other than the Trauma ICU (Unit A), shall be transferred to the Trauma ICU (Unit A) as soon as possible. *Phase II* All MERS-CoV patients will be cohorted in one unit. If the number of patients exceeds its capacity, then other units are identified to receive the additional cases
Closure of all nonessential hospital functions	*Phase I* All services run without interruptions except for certain precautions for MERS patients *Phase II* All elective surgery shall be canceled to free more ICU beds *Phase III* All elective cardiac surgery shall be canceled Outpatient clinic visits shall be limited to urgent visits only
Healthcare worker (HCW) management	All HCWs shall be aware of 1. Relevant infection prevention and control policies and procedures 2. Their annual influenza immunization status. If not vaccinated, please contact the employee health clinic to arrange for an appointment 3. Their N95 fit check/test status. If have not been fit-tested, please contact the employee health clinic to arrange for an appointment HCWs exposed to a confirmed MERS-CoV case shall be assessed according to a predetermined protocol HCWs requiring isolation at home and happen to share a room with another HCW will be provided a room in the designated accommodation for isolation till cleared by the Infection Prevention and Control Department An Infection Prevention and Control officer is available 24 h per day, 7 days per week

*ICU* intensive care unit, *HCW* health care worker, *MERS-CoV* Middle East respiratory syndrome coronavirus

An Infectious Disease Epidemic Plan (IDEP) was initially released in May 2014 and was revised in March 2015. Table 1 describes selected plan elements. According to the IDEP, one unit (Unit A) was designated as the primary receiving unit for MERS patients, because of its geographic location being away from main hospital traffic and because 7 of its 8 rooms were negative-pressure airborne infection isolation rooms (AIIR). The plan was not explicit about which units would be used if the number of cases exceeded the capacity of this unit. However, unit B had 4 negative-pressure rooms.

### Management of MERS patients

In our hospital, MERS was suspected based on clinical presentation and confirmed by laboratory testing as recommended by the WHO and the Saudi Ministry of Health [4, 13]. In our ICU, the workup of patients having lower respiratory tract infections was standardized to include bacterial gram stain and culture, MERS-CoV polymerase chain reaction (PCR), H1N1 PCR, bacterial and viral multiplex PCR on respiratory samples, mycoplasma, chlamydia and legionella serology and, if suspected, tuberculosis workup. The respiratory samples were routinely obtained by blind deep tracheal aspirate in intubated patients. In the hospital biosafety level 2 laboratory, MERS-CoV screening was performed by real-time reverse-transcription (rRT) PCR on respiratory samples by checking for the upstream E protein genome (Roche Modular Dx Coronavirus) and infection confirmation by detecting the open reading frame 1a genome (MERS-CoV kit from TIB Molbiol) [14]. Laboratory workers were fit-tested and applied N95 respirators while handing respiratory samples. Positive samples were sent to the Saudi Ministry of Health reference laboratory for confirmation. Viral culture was not performed as biosafety level 3 is needed.

### Data collection

We noted MERS cases admitted to the ICU from July 1 to October 21, 2015, and collected data on the clinical characteristics, management and outcomes of the affected HCWs. We also obtained data about the rRT-PCR performed for ICU patients. We identified the physicians and nurses who reported sick leave for respiratory illnesses. As surrogate for infection control practices, we obtained data on the related consumables for Units A and B before and during the outbreak (April–September 30, 2015). We also collected qualitatively our own observations and those of other HCWs on the ICU response during the outbreak using interviews and open discussions.

### Statistical analysis

Descriptive data were presented as means and standard deviations or frequencies and percentages, as appropriate. The infection prevention and control consumables were compared in the 4 months before (April to July) and the 2 months during (August and September) the outbreak.

## Results

### Description of the MERS outbreak

**Fig. 1:**
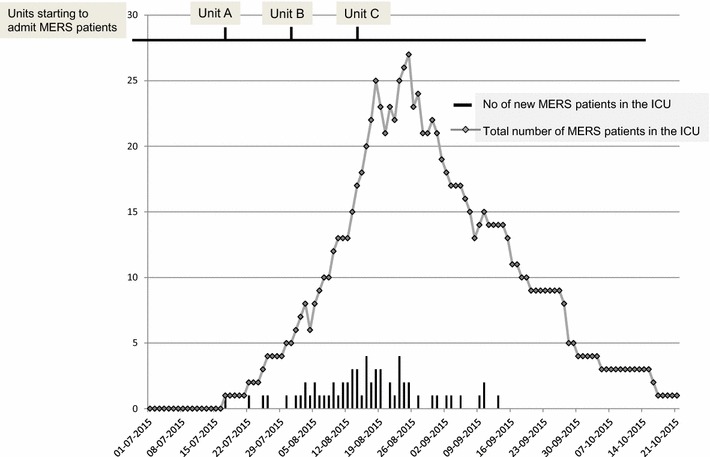
Time line of MERS cases in all our units between July 1 and October 21, 2015

In July 2015, five cases of acute respiratory failure were referred to the ICU team from the ED and wards and were diagnosed to have MERS pneumonia. As the number of MERS patients increased, the IDEP was activated on August 2, and included strict implementation of infection control measures, including airborne and contact isolation for confirmed and probable MERS cases, and droplet and contact isolation for suspected cases [8]. On August 18, Phase III of the IDEP was activated (Table 1), which included ED closure, elective surgical procedure cancelation and outpatient clinic suspension [8]. Meanwhile, the ICU maintained full operations. Figure 1 shows the time line of MERS cases in all our units between July 1 and October 21, 2015. During the outbreak, we received a total of 63 MERS cases with the peak being on August 25, 2015, when the census of confirmed MERS cases reached 27 in the three MERS-designated ICUs. The last new ICU case was on September 13, 2015.

#### Diagnosing MERS

Suspected MERS cases had rRT-PCR on nasopharyngeal swabs in nonintubated patients and on deep tracheal aspirates in intubated patients. Fiberoptic bronchoscopy was not performed for the diagnostic workup of MERS or ventilator-associated pneumonia. Repeated testing was frequently needed to make the diagnosis. Among our 63 critically ill MERS patients, the initial MERS-CoV testing was performed on nasopharyngeal swabs in 29 and deep tracheal aspirates in the rest (n = 34). In the first sample, MERS-CoV was detected in only 6/29 (20.7 %) nasopharyngeal samples and 26/34 (76.5 %) deep tracheal aspirates. After initial negative or equivocal nasopharyngeal swabs (n = 23), a second nasopharyngeal swab was performed in 16 patients (positive in 37.5 %) and deep tracheal aspirate in 5 (positive in 100 %). Our microbiology laboratory extended its working hours and prioritized testing samples coming from the ICU and the rest of the hospital. The number of MERS-CoV tests performed on ICU patients went up from an average of 1.5 before to 5.3 per day during the outbreak with a maximum of 19 tests on September 4, 2015. In patients with suspected MERS and negative rRT-PCR, the test was repeated after 1–2 days. There was a general consensus among our intensivists that three negative lower respiratory samples and low clinical pretest probability were needed to exclude MERS-CoV infection. For patients with confirmed MERS, the test was repeated twice weekly until 3 consecutive tests were negative.

#### Characteristics, management and outcomes of MERS patients

The mean age of the 63 patients was 57.9 ± 18.6 years with the majority (69.8 %) being males. Eight hospital workers required ICU admission after acquiring MERS. Table 2 summarizes their characteristics. Half of them did not have direct contact with patients. One of them was a pregnant nurse that worked in the ED. All but one required intubation.

**Table 2:** Characteristics, management and outcomes for healthcare workers (n = 8) infected with MERS-CoV and admitted to the intensive care unit during the outbreak

Job	Patient 1	Patient 2	Patient 3	Patient 4	Patient 5	Patient 6	Patient 7	Patient 8
	Ward nurse	Ward nurse	Clinic nurse	Physician	RT technician	ED transporter	Executive	AC technician
*Clinical presentation*								
Dyspnea	Yes	Yes	Yes	Yes	Yes	Yes	Yes	Yes
Body temperature	39.4 °C	38.9 °C	37.9 °C	38.3 °C	39.5 °C	39.4 °C	39.0 °C	38.9 °C
WBC × 10^9^/L	11.6	13.1	3.5	5.9	5.1	6.0	3.7	6.8
Shock	Yes	Yes	Yes	No	Yes	Yes	Yes	Yes
PaO_2_/FIO_2_	71	91	56	453	64	100	107	126
CXR findings	Bilateral lower lobe infiltrates	Right lower lobe infiltrates	Diffuse bilateral infiltrate	Bilateral lower lobe infiltrates	Right lower lobe infiltrates	Diffuse bilateral infiltrates	Left lower lobe infiltrates	Left lower lobe infiltrates
*Management*								
Noninvasive ventilation	Yes (failed)	No	No	No	No	No	Yes (failed)	No
Intubation	Yes	Yes	Yes	No	Yes	Yes	Yes	Yes
ARDS management	Cisatracurium infusion; prone positioning	Cisatracurium infusion; prone positioning; inhaled nitric oxide	Cisatracurium infusion	NA	Cisatracurium infusion	Cisatracurium infusion	Cisatracurium infusion; prone positioning; inhaled nitric oxide	Cisatracurium infusion; prone positioning
Vasopressors	Yes	Yes	Yes	No	Yes	Yes	Yes	Yes
CVVHD (duration)	No	Yes (26 days)	No	No	No	Yes (6 days)	Yes (37 days)	Yes (47 days)
ECMO (duration)	No	Yes (15 days)	No	No	No	No	No	No
Tracheostomy	No	No	No	No	No	No	Yes (surgical; on day 25 after intubation)	Yes (surgical; on day 30 after intubation)
*Outcomes*								
MV duration (days)	20	26	13	0	13	6	33	57
ICU LOS (days)	28	28	17	3	16	7	56	63
Hospital LOS (days)	44	42	31 days	7	22	26	66	101
Vital status at hospital discharge	Survived	Survived	Survived	Survived	Survived	Survived	Survived	Survived

*WBC* white blood cells, *PaO*_*2*_ */FiO*_*2*_ ratio of arterial oxygen partial pressure to fractional inspired oxygen, *CXR* chest X-ray, *MV* mechanical ventilation, *ARDS* acute respiratory distress syndrome, *CVVHD* continuous venovenous hemodialysis, *ECMO* extracorporeal membrane oxygenation, *ICU* intensive care unit, *LOS* length of stay, *RT* respiratory therapist, *ED* emergency department, *AC* air condition

The medical management of MERS patients was largely supportive. Most (80.9 %) MERS patients required endotracheal intubation, which was performed by the most experienced available physician with airborne precautions. Lung-protective ventilation was implemented for acute respiratory distress syndrome with tidal volumes (4–6 ml/Kg of ideal body weight). To reduce the airborne generating procedures, we discouraged the use of noninvasive ventilation for suspected MERS cases. Nevertheless, it was used in the initial management of 12 (19.0 %) patients. These patients were either suspected to have concomitant cardiogenic pulmonary edema or had milder disease. Intubation was needed for 10 patients. High-flow oxygen therapy was not used as it was unavailable. When needed, bronchodilators were used via metered dose inhalers rather than nebulizers. Most (58.7 %) MERS patients required vasopressors. Renal replacement therapy was provided for 37 (58.7 %) patients.

For the hospital workers acquiring MERS (n = 8), cisatracurium infusion was used in 7 (87.5 %), early prone positioning in 4 (50 %), continuous renal replacement therapy in 4 (50 %) and extracorporeal membrane oxygenation in 1 (Table 2). None of the patients received ribavirin, interferon therapy or high-dose steroids.

The hospital mortality of MERS patients was 63.4 % with all deaths occurring in the ICU. All hospital workers who had MERS survived and were discharged to home. The ICU and hospital length of stay were prolonged (15.0 ± 17.0 and 30.6 ± 23.1 days, respectively).

### The ICU outbreak management

#### Leadership and communication

During the outbreak, our hospital established a Command Center, which met twice daily, and oversaw all interventions in accordance to the IDEP. The Intensive Care Department chairman was a member of the Command Center and presented daily the number of suspected and confirmed cases in the ICU, bed and staff management issues and any challenges. The Department chairman attended all morning handover meetings in the ICU where he received input from the ICU teams and provided feedback from the Command Center.

The hospital provided an intranet page that had educational material on MERS, MERS management guidelines and proper infection control practices. The page was regularly updated. Additionally, the hospital frequently informed staff about the MERS outbreak status through emails. Staff could communicate with the Command Center regarding any outbreak-related concern or question. The Intensive Care Department communicated with the medical staff about the Saudi Ministry of Health and WHO practice guidelines. Before and during the outbreak, the WHO interim guidance for the management of suspected and confirmed MERS-CoV infection [15] was circulated to our ICU staff. Moreover, a letter expressing gratitude and encouragement was sent from the Department Chairman to all HCWs.

Family visiting to MERS patients was restricted from an open visiting policy to 2 h per day during the evening with visitors not allowed to enter patient rooms. Visiting outside these hours was allowed in selected cases if the clinical condition required. To update the patients’ families, the ICU consultant contacted and updated the next of kin by phone every day and addressed the family concerns. A nurse was assigned to screen all staff and visitors entering each unit by asking for symptoms of acute respiratory infection and measuring temperature. Staff and family members with symptoms of acute respiratory illness or fever were not allowed to enter.

#### Facility management

The initial MERS cases were admitted to the designated MERS unit (Unit A). As the number of patients increased, the ICU leadership identified other ICUs as potential placement units. The hospital clinical engineers converted a total of 24 standard rooms in Unit B and Unit C to negative-pressure rooms by increasing air exhaust more than supply by 50 cubic feet per minute. As the number of suspected and confirmed MERS patients increased, Unit B and then Unit C were used.

#### Disposition of non-MERS patients

As the number of our MERS patients increased beyond Unit A capacity, patients without MERS were transferred to other units or hospitals to increase bed capacity. The old pediatric ICU, which was recently vacated in June 2015 after the opening of a new pediatric hospital, was used to care for stable and long-term patients. During the outbreak, 13 and 7 patients from Units A and B, respectively, were transferred to the other ICUs (Units C-E and the old pediatric ICU), and 1 patient to another hospital.

#### Staffing

The care for MERS patients was demanding. For example, 4 of the 8 affected HCWs required prone positioning for the management of acute respiratory distress syndrome (Table 2). Also 4 of them required continuous renal replacement therapy for up to 37 days (Table 2). The care was also associated with significant exposure risk. This can be reflected in the duration of mechanical ventilation for the HCWs who required intubation (7–57 days) and length of ICU stay (7–63 days) (Table 2). During the outbreak, the nurse-to-patient ratio was mostly 1:1 except for one patient on ECMO (2:1). Additionally, 1–2 additional nurses were deployed in each unit to assist in procedures such as prone positioning and to monitor and correct infection control practices. In unit A, for instance, the nurse-to-patient ratio was 1:1.2 before the outbreak and became 1:0.8 during the outbreak. We restricted medical management to the attending physicians, senior registrars and critical care fellows. Rotating residents were not allowed to work in the ICU during the outbreak. Entry of nonclinical staff, such as research coordinators, was restricted and the ongoing clinical trials were put on hold, except for MERS studies, to reduce staff exposure.

During the outbreak, concerns among ICU staff were raised about acquiring MERS and transmitting the virus to their families; a concern that was substantiated by seeing hospital workers infected and developing critical illness. However, many felt privileged to be part ICU team managing the outbreak and taking care of MERS patients; none refused to report to work as per schedule. Two pregnant ICU nurses were redeployed to low-risk units. Staff members who developed fever, respiratory symptoms or gastrointestinal illness were asked not to present to work, but rather to report to the ED or the Employee Health Clinic depending on their illness severity. Of the 152 bedside nurses covering Units A and B, 41 (27.0 %) nurses had symptoms of acute respiratory infection during the outbreak and consequently had nasopharyngeal swabs obtained for MERS-CoV; all tested negative. Their total sick leave duration was 138 days (range: 1–15 days per nurse). In comparison, in the 2 months before the outbreak, 26 (17.1 %) nurses had sick leaves for a total of 33 days (range 1–2 days per nurse). Of the 49 nonresident ICU physicians, 9 (18.4 %) physicians received sick leave for a total of 30 days (range 3–4 days per physician). In Unit C, two nurses (1.0 %) of 196 nurses who worked in MERS units (Units A, B and C) and one rotating resident (1.3 %) out of 80 physicians covering the ICUs tested positive for MERS-CoV. The resident and one of the nurses were symptomatic and required hospitalization in a MERS ward for approximately 1 week and both recovered fully.

### Logistics for infection prevention and control

In February 2012, long before the MERS outbreak, droplet precautions had been added to the standard precautions for all ICU patients, mainly to prevent the transmission of influenza. During the outbreak, airborne precautions were added for all confirmed and suspected MERS cases. Although all staff were required to be fit-tested for the N95 respirators before the outbreak (Table 1), we discovered that many ICU staff were not tested. During the outbreak, a clinic was emergently opened to fit test HCWs and the results were documented.

Specific policies and procedures were developed or updated for donning and doffing personal protective equipment (PPE). Related visual instructions were provided inside each ICU room. Outside patient rooms, carts containing PPE were organized to facilitate donning in the correct sequence. During the outbreak, additional training on hand hygiene techniques and PPE application was provided. Housekeepers were also retrained on proper cleaning techniques and PPE use. The Intensive Care Department worked closely with the Infection Prevention and Control Department on all aspects of infection control.

The implementation of such infection control measures required having adequate PPE supplies, such as respirators, goggles, face shields and gowns. Table 3 describes the consumption of surface disinfectants, antiseptic alcohol for hand hygiene, N95 masks and other PPE before and during the MERS-CoV outbreak. During the outbreak, the consumption of detergent surface disinfectant and ethyl alcohol for alcohol-based hand rub increased by almost 30 % and the use of N95 masks increased by >15 times compared to the preceding 4 months. The number of examination gowns per patient per day decreased during the outbreak probably due to staff avoiding unnecessary exposure. Twenty-four powered air-purifying respirators were made available to staff who failed the N95 respirator fit test. They were used by 8 physicians, 7 nurses and 14 respiratory therapists. Training sessions on their application were conducted.

**Table 3:** Consumption of 2 % chlorhexidine for surface disinfection, antiseptic ethyl alcohol for hand hygiene, N95 respiratory masks and other personal protective equipment before and during the MERS-CoV outbreak

	Before the outbreak (4 months: April–July)	During the outbreak (2 months: August–September)
Detergent disinfectant (mL/ICU room/day)	23.0	34.2
Nonalcoholic surface disinfectant wipes		
Total number consumed	10,300	11,350
Number consumed per room per day	2.9	6.4
Average volume of alcohol-based hand rub (mL/patient/day)	120	163
N95 masks		
Total number consumed	3610	29,605
Number consumed per patient per day	1.0	16.7
Gowns (regular nonsterile disposable)		
Total number consumed	179,100	7000
Number consumed per patient per day	50.6	4.0
Gowns (waterproof nonsterile disposable)		
Total number consumed	1015	23,916
Number consumed per patient per day	0.3	13.5
Eye-protective glasses		
Total number consumed	190	10,180
Number consumed per patient per day	0.05	5.75
Examination gloves, *N*		
Total number consumed	554,600	187,900
Number consumed per patient per day	156.8	106.2

## Discussion

In this report, we described how the ICU responded to a MERS-CoV outbreak at a tertiary-care hospital. The outbreak led to 63 MERS patients requiring prolonged ICU care and most received invasive mechanical ventilation, vasopressors and renal replacement therapy. The overall mortality was 63 %, but all affected hospital workers survived. The outbreak management included almost tripling the ICU capacity of negative-pressure rooms and intensifying infection prevention and control practices. Even though ICU staff had significant exposure risk, very small number acquired MERS-CoV.

Response to incidents such as an infectious hospital outbreak requires a robust hospital-wide command and control structure that is able to make rapid informed decisions across an institution. As it was the case in our hospital, the control of outbreak may require major interventions such as closing the ED, suspending elective surgeries, preventing inter-facility patient transfers, canceling ambulatory clinics and outpatient diagnostic procedures, preventing hospital staff from working at other institutions and restricting hospital visitors [16]. Our hospital had a preexisting IDEP, which facilitated managing and containing the MERS-CoV outbreak. Such a plan is mandatory for every hospital.

MERS infection is associated with several challenges. Its presenting symptoms overlap with those of other severe acute respiratory illnesses and include fever (71 %), cough (68 %), dyspnea (66 %) and diarrhea (32 %) [17], often in older adults with preexisting chronic comorbidities [17, 18]. Common laboratory abnormalities include leukopenia, lymphocytopenia, thrombocytopenia and elevated serum creatinine, lactate dehydrogenase, and liver enzymes [17]. The initial chest radiographs show minimal abnormality to extensive bilateral infiltrates [17]. Unfortunately, many frontline physicians are unfamiliar with the MERS case definition, probably because cases are sporadic, leading to delayed or even missed diagnosis. Delayed recognition may lead to exposing many other patients, visitors and HCWs to the infection as it was the case in our hospital. MERS-CoV nosocomial transmission is thought to be via respiratory droplets, and contact spread is suspected [19]. In Korea, the delayed diagnosis of an infected traveler to the Arabian Peninsula led to 186 MERS cases and resulted in intra- and inter-hospital transmission [20]. In Saudi Arabia, 45 % of MERS cases were acquired in the healthcare setting with 12 % of all cases being HCWs [7]. Therefore, taking a detailed history, knowing MERS case definitions, standardizing pneumonia workup, obtaining lower respiratory tract specimens [21] and implementing droplet isolation for suspected cases are crucial interventions to break the transmission chain in the healthcare setting.

Admitting MERS patients in single-bedded negative-pressure rooms and cohorting them in selected units are recommended to facilitate providing care and monitoring [22]. During an outbreak, clinical engineering should have expedient plans to convert standard rooms. Retrofitting the rooms with externally exhausted HEPA filters may be a solution [16]. Outbreaks can lead to significant increase in the need for ICU beds, but may simultaneously reduce the available beds. The SARS outbreak in Toronto led to 10-day closures of 35 ICU beds, which represented 38 % of the tertiary-care university medical-surgical ICU beds in Toronto [16]. Hence, hospitals should always have plans to augment ICU bed capacity, such as by transforming general wards. The ability of any hospital to deal with an infectious outbreak is decided by the availability of ICU beds [23].

Caring for MERS patients represents a substantial exposure risk for ICU staff because of three reasons: high exposure dose, long daily contact hours and prolonged ICU stay with viral shedding. MERS-CoV patients requiring ICU admission have higher viral load than other MERS patients [24]. Aerosol-generating procedures, such as noninvasive ventilation, suctioning and bronchoscopy, add to the exposure and transmission risk [25]. Extended bedside care is needed for MERS patients due to the requirement of organ support such as mechanical ventilation, vasopressor therapy, continuous renal replacement therapy, prone positioning and extracorporeal membrane oxygenation [2, 3]. In this study, we observed an increase in the nurse-to-patient ratio from 1:1.2 to approximately 1:0.8 during the outbreak. The SARS epidemic was also associated with increases in the nurse-to-patient ratio [26]. Stay in the ICU can last for weeks [3], which we observed. Additionally, MERS-CoV shedding can be prolonged and may last for >30 days [27].

The exposure risk to MERS-CoV can exert significant psychosocial stress on HCWs. Death has occurred in young HCWs who acquired MERS-CoV infection [28], which adds to this fear. During the SARS outbreak in Toronto, a survey of HCWs found that >60 % of respondents reported SARS-related concern for their own or their family’s health [29]. Moreover, 29 % of respondents had probable emotional distress [29]. During our outbreak, many ICU staff expressed concerns about acquiring MERS. Staff safety should be a primary goal in a hospital infectious outbreak. Pregnant and immunocompromised staff should be redeployed to lower-risk areas [30], which we did. Proper exposure management should be pre-planned, which was determined in our IDEP. N95 respirator fit testing should be performed at hiring of new staff and done for all other staff before any outbreak. Although fit testing was required for all staff, many did not have fit testing done. However, in response to the outbreak, fit testing was performed to all staff and powered air-purifying respirators were provided to those who failed fit testing. Strict infection prevention and control practices should be implemented and audited. This was performed in our units. Repetitive training is recommended [31]. Despite intensive infection prevention practices, 3 of our ICU staff had MERS-CoV infection during the outbreak likely due to suboptimal PPE use while intubating yet undiagnosed patients.

MERS management was supportive and largely adhered to the WHO recommendations [15]. It included intubation, early prone positioning and neuromuscular blockade for moderate-to-severe acute respiratory distress syndrome as these interventions have been shown to improve the outcomes of ARDS patients [32, 33]. Although noninvasive ventilation use was discouraged, it was used in 12 patients. The WHO considers noninvasive ventilation an option in selected MERS cases [15]. It should be used as a short trial without delaying intubation if unsuccessful [15]. Moreover, high-flow oxygen by nasal cannula may be another option [15, 34]; however, the associated transmission risk as a result of aerosol generation is unknown. Systematic corticosteroids, ribavirin and interferon were avoided as they have no proven benefit [15, 35]. Our MERS patients had high mortality (63 %). The previously reported mortality of MERS patients who had critical illness ranged from 58 to 84 % [2, 3, 18]. None of our HCWs who developed MERS died, which was gratifying to our staff.

## Conclusions

Our MERS-CoV hospital outbreak stressed our system to unprecedented limits. We learned many lessons from it (Table 4). The successful management of outbreak required integrating ICU functions with the hospital-wide plans, having preparedness plans, implementing proper infection control practices and managing staffing and staff exposure.

**Table 4:** Lessons learned from the MERS-CoV outbreak

Every hospital should have an Infectious Disease Epidemic Plan that should govern the response to an infectious disease outbreak. The response should cover organizing patient services, implementing infection control, managing employee exposure and communicating with national health services and with hospital staff Hospital leaders should be prepared to increase the capacity of negative-pressure airborne infection isolation rooms in the case of an infectious disease outbreak All healthcare workers should receive training on proper hand hygiene and personal protective equipment application. Hand hygiene and personal protective equipment practices should be monitored. Education should be repeated periodically All healthcare workers should be fit-tested for N95 respirators on hire with the result documented in their files. Periodic audit of this requirement should be done Hospitals should make plans to acutely increase personal protective equipment supplies as consumption increases tremendously during an infectious disease outbreak Hospital and ICU leaders should have plans to cover healthcare workers who are exposed or become symptomatic to avoid potential staff shortage	

## Abbreviations

ED: Emergency Department
HCW: healthcare worker
ICU: intensive care unit
IDEP: Infectious Disease Epidemic Plan
MERS: Middle East respiratory syndrome
PPE: personal protective equipment
rRT-PCR: real-time reverse-transcription polymerase chain reaction
WHO: World Health Organization
